# Global control of DNA replication timing by the budding yeast telomere protein Rif1

**DOI:** 10.1186/1756-8935-6-S1-P99

**Published:** 2013-03-18

**Authors:** Laure Lemmens, Stefano Mattarocci, Isabella Marcomini, Tianlai Shi, Cindy Follonier, Massimo Lopes, Nicolas Thomä, David Shore

**Affiliations:** 1 NCCR “Frontiers in Genetics” program and Department of Molecular Biology, University of Geneva, Geneva 4, CH-1211, Switzerland; 2 Friedrich Miescher Institute for Biomedical Research, Basel, CH-4058, Switzerland; 3 Institute of Molecular Cancer Research, University of Zurich, Zurich, CH-8057, Switzerland

## Background

DNA replication in eukaryotes is initiated from specific chromosomal sites (origins) that fire in a defined, cell type-specific temporal pattern. This replication program appears to be under epigenetic control through mechanisms that are still poorly understood.

## Materials and methods

Studies were done in *Saccharomyces cerevisiae* (W303 background) using standard genetic and molecular methods. Chromatin immunoprecipitation assays were performed in strains in which genes encoding DNA polymerase 1 or 2 were epitope-tagged at their endogenous loci.

## Results

We showed previously that telomere TG-repeat tract length exerts an epigenetic effect in *cis* on the activity of nearby subtelomeric replication origins, such that a shortened telomere will replicate earlier [1]. Here we show that deletion of the *RIF1* gene, which encodes a telomere-specific Rap1-interacting protein involved in telomere length regulation and telomere “capping” [2-5], also leads to premature replication of two different subtelomeric regions examined. A similar effect of *RIF1* deletion on other subtelomeric regions has recently been described [6]. We show here that the effect of *RIF1* deletion is epistatic to loss of Tel1 or Mec1 (ATM and ATR kinases), does not affect the intra-S phase checkpoint, and operates through a different pathway than the silencing protein Sir3. Deletion of a normally dormant telomere-proximal replication origin exerts a similar effect on replication timing as does deletion of *RIF1*, and these two effects are additive. Strikingly, deletion of *RIF1* partially suppresses temperature-sensitive mutations in a number of essential genes that encode regulators of DNA replication initiation, without affecting the levels of the relevant gene products, several of which are present in limiting amounts.

## Conclusions

The budding yeast telomere-binding protein Rif1 is shown here to be a global regulator of DNA replication initiation whose loss leads to precocious replication of subtelomeric domains in the budding yeast. This appears to be a highly conserved function of Rif1, since its homologs have recently been shown to exert related effects in both fission yeast and mammalian cells [7-9]. Experiments will be described aimed at understanding the mechanistic basis of the effect of Rif1 on replication timing.
